# The reverse halo sign in emergency chest CT: A diagnostic guide across diverse pulmonary conditions

**DOI:** 10.1007/s10140-025-02337-2

**Published:** 2025-04-15

**Authors:** Furkan Ufuk, Iclal Ocak, Lydia Chelala, Luis Landeras

**Affiliations:** Department of Radiology, The University of Chicago Medicine, Chicago, IL 60637 USA

**Keywords:** Atoll sign, Infection, Organizing pneumonia, Malignancy, Pulmonary Embolism, Trauma

## Abstract

The atoll sign, also known as the reverse halo sign (RHS), is defined by a central ground-glass opacity encircled by a rim of consolidation. Originally described in the context of organizing pneumonia, it is now recognized across a spectrum of conditions—including infectious, inflammatory, vascular, malignant, and traumatic pathologies. This pictorial essay examines the various clinical scenarios in which the RHS is observed on emergency CT, highlighting its crucial role in differential diagnosis and patient management.

**Supplementary Information:**

The online version contains supplementary material available at 10.1007/s10140-025-02337-2.

## Introduction

**Fig. 1:**
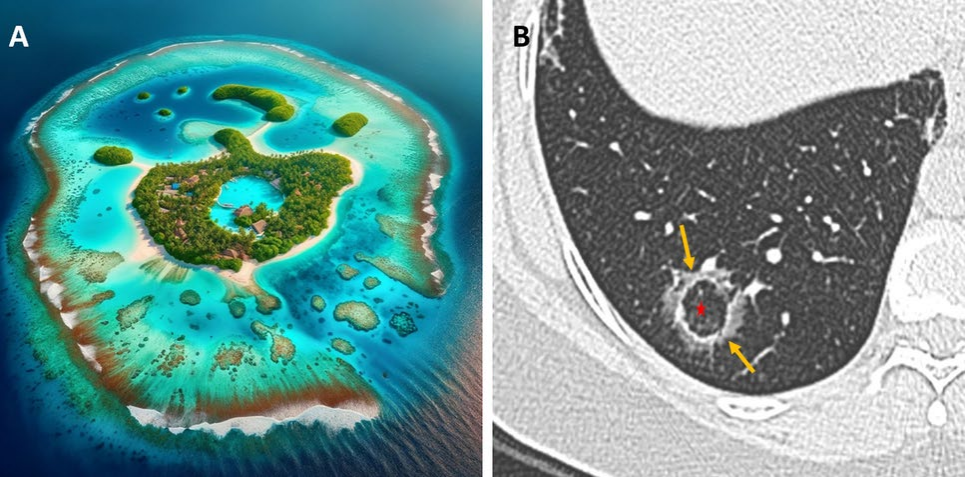
**A** Atoll: A ring-shaped coral reef or island. **B** Atoll Sign: The central area of ground-glass attenuation represents alveolar septal inflammation (Star), while the peripheral consolidation corresponds to inflammatory tissue (arrows)

The reverse halo sign (RHS), also known as the “atoll sign,” is defined by a central area of ground-glass opacity (GGO) encircled by a denser rim of consolidation (Fig. 1) [1]. Increased awareness of the RHS has shown that a wide range of pulmonary conditions can present with this pattern [1–3]. In the emergency setting, accurately distinguishing these entities on chest CT requires integration of imaging findings with the patient's clinical presentation, laboratory data, immune status, and relevant history. This article provides an overview of the major causes of the RHS and essential CT findings.

### Infectious causes

#### Bacterial pneumonia

**Fig. 2:**
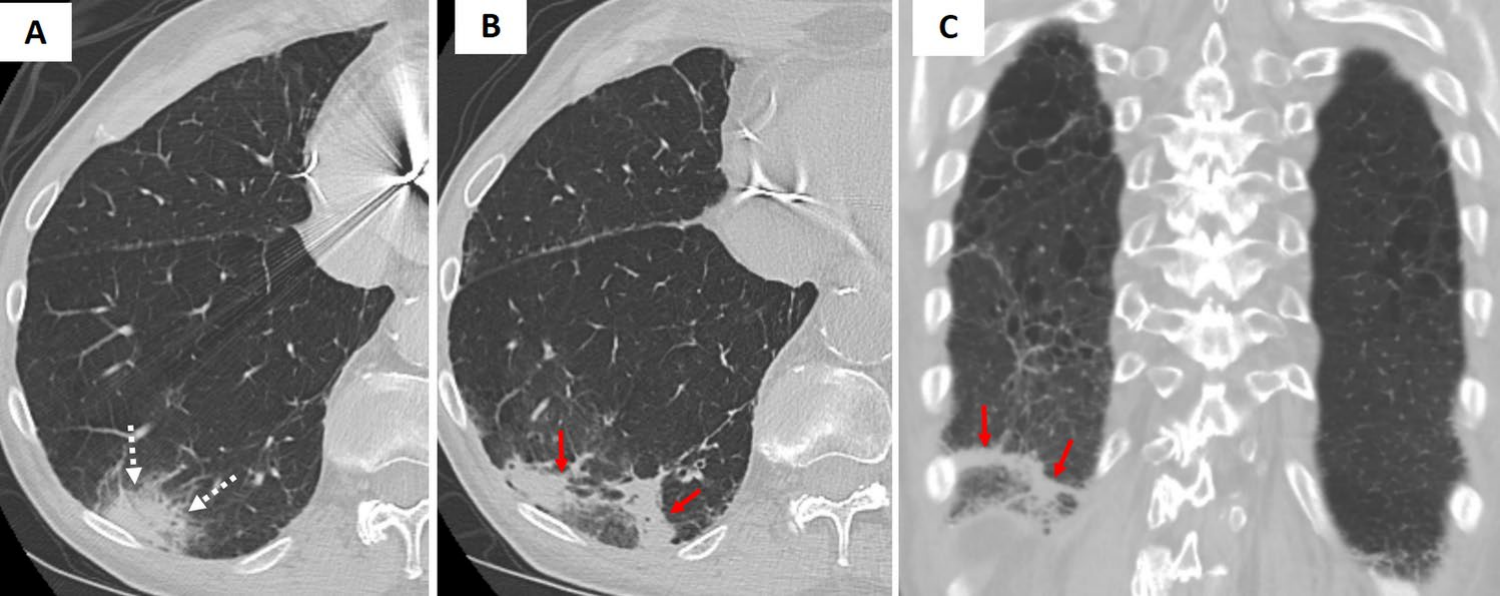
**A** Initial axial chest CT image demonstrates focal consolidation within the right lower lobe in a patient with COPD presenting with fever and cough. Sputum culture confirmed *Streptococcus pneumoniae* infection. **B** Axial and (**C**) coronal chest CT images obtained two weeks later reveal the development of the reverse halo (atoll) sign at the site of the prior consolidation

**Fig. 3:**
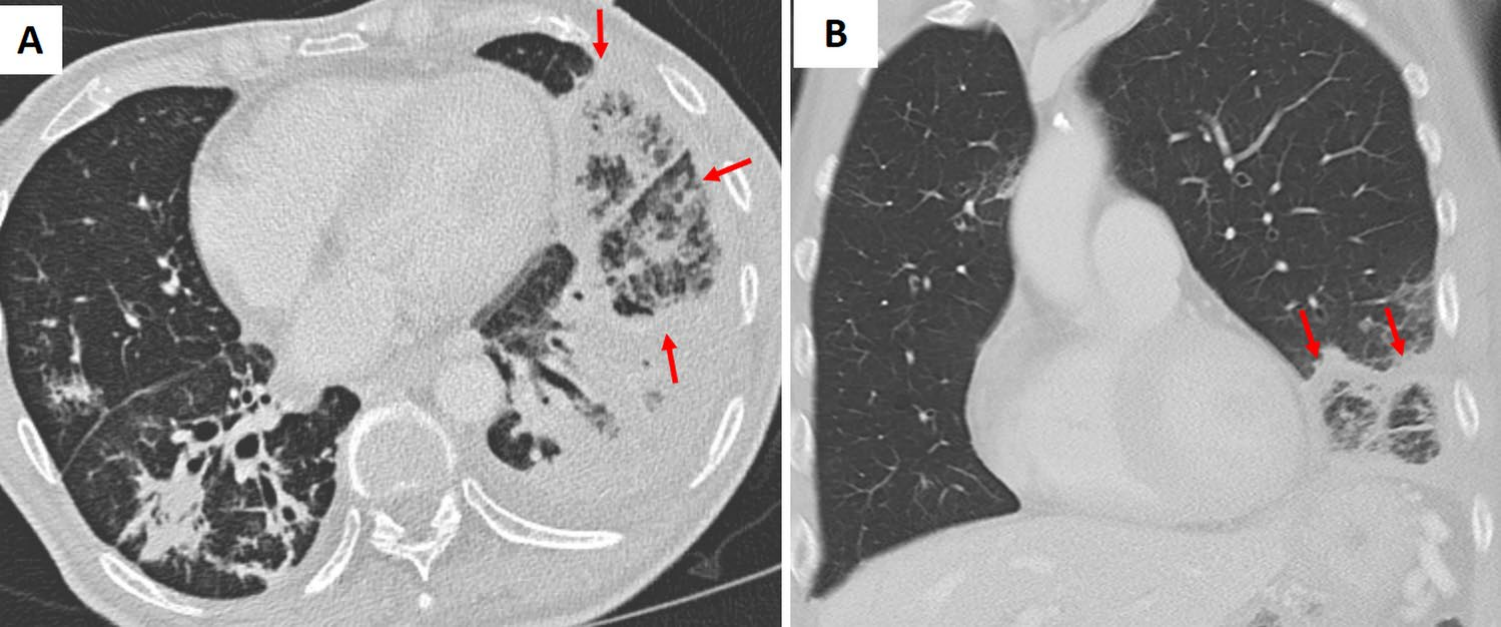
**A** Axial and (**B**) coronal chest CT images of a patient with oral squamous cell carcinoma and immunosuppression demonstrate bilateral lower lobe and lingular consolidative pulmonary opacities. The left lower lobe and lingular opacities exhibit the reverse halo (atoll) sign (arrows). Sputum culture confirmed *Staphylococcus aureus* infection

Bacterial pneumonia may manifest a RHS during the organizing phase of lung injury. Histopathologically, the central GGO corresponded to residual inflammatory exudates, while the peripheral rim of consolidation reflected the ongoing organization of the infection. Bacterial pneumonia can be distinguished from other causes of the RHS by integrating the patient's clinical context with its non-specific CT features. In bacterial pneumonia, including tuberculosis, the RHS typically appears during the evolving phase of the infection, characterized by a smooth, uniform rim of consolidation with surrounding GGO and ipsilateral pleural effusion (Figures 2 and S1). However, imaging findings may vary based on the patient’s immune status (Figure 3) [4].

#### Fungal infections

**Fig. 4:**
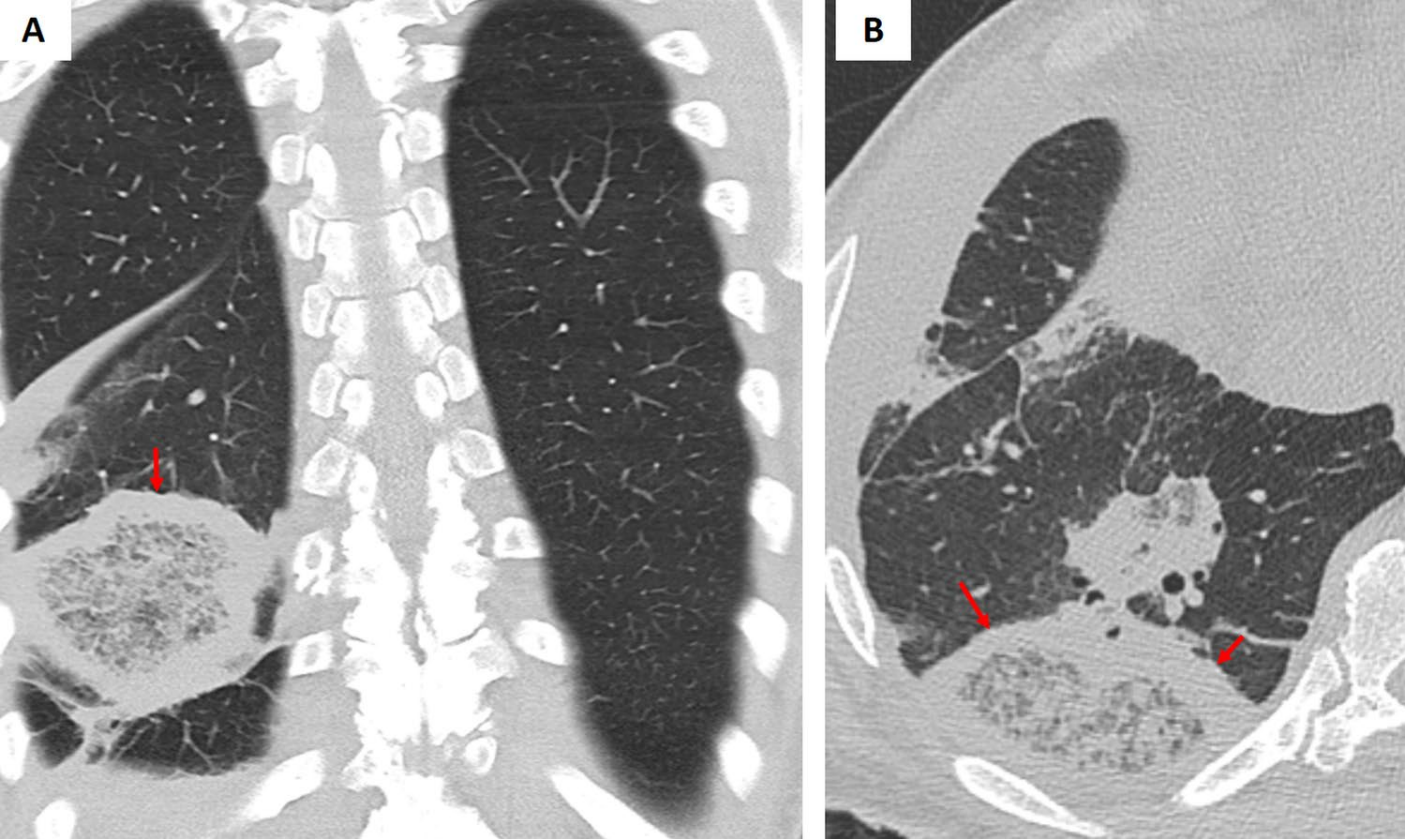
**A** Coronal and (**B**) axial chest CT images of a patient with acute myeloid leukemia (AML) demonstrate a right lower lobe peripheral pulmonary opacity with a bird’s nest sign (arrows). This finding represents a reverse halo sign characterized by irregular and intersecting lines or stranding within an area of ground-glass opacity, indicative of invasive fungal infection. The patient was subsequently diagnosed with mucormycosis

Fungal pathogens such as mucormycosis and aspergillosis can give rise to the so-called “bird’s nest” variant of the reversed halo (atoll) sign [1–4]. The “bird’s nest” pattern in fungal infections is marked by irregular, intersecting lines within the central area (Fig. 4). These opportunistic infections are particularly prevalent in immunocompromised or diabetic patients. In mucormycosis, the central GGO with intersecting lines often represents necrotic tissue, while the peripheral rim corresponds to inflammatory or fibrotic changes. Early detection of this pattern is critical because mucormycosis requires urgent and aggressive antifungal therapy, and delays in diagnosis can have dire consequences. Although the imaging appearance of aspergillosis may overlap with that of mucormycosis, subtle differences along with clinical cues (including specific risk factors and serologic tests) can help distinguish these entities [3, 4].

#### Viral Pneumonia

**Fig. 5:**
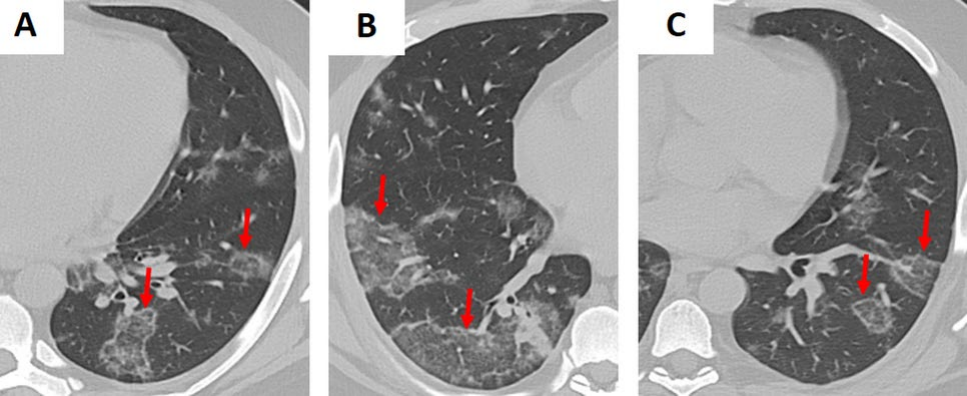
Examples of bilateral, multifocal atoll sign (arrows) in patients with (A–C) influenza pneumonia (*H1N1*). The images demonstrate multiple areas of ground-glass opacity surrounded by a peripheral rim of consolidation, characteristic of the reverse halo sign

**Fig. 6:**
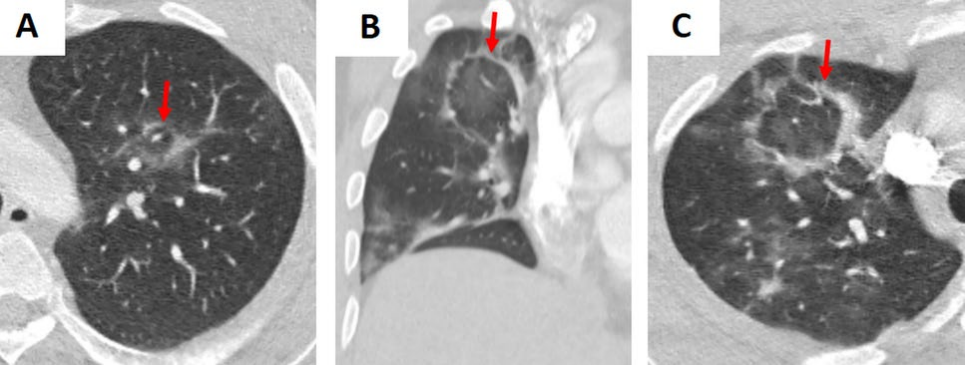
Examples of the atoll sign (arrows) in a patient with (**A–C**) COVID-19. The images demonstrate multiple areas of ground-glass opacity (GGO) surrounded by a peripheral rim of consolidation, characteristic of the reverse halo sign. Note the presence of scattered areas of GGO throughout the lungs

A variety of viruses—including influenza, respiratory syncytial virus (RSV), cytomegalovirus (CMV), and SARS-CoV-2 (COVID-19)—may demonstrate the RHS on CT imaging (Figs. 5, 6, and S2) [4, 5]. In viral pneumonia, the central GGO reflects inflammatory changes triggered by viral injury, while the surrounding consolidation indicates the host immune response. For example, in COVID-19 pneumonia, the RHS typically appears during the later or resolving stages of infection and may signal the development of post-inflammatory fibrosis [5]. Unlike bacterial or fungal infections, viral pneumonia is usually accompanied by a clinical history of recent viral exposure, fever, and cough, with laboratory findings that often include lymphopenia rather than the neutrophilia seen in bacterial infections [3–5].

#### Parasitic infections

**Fig. 7:**
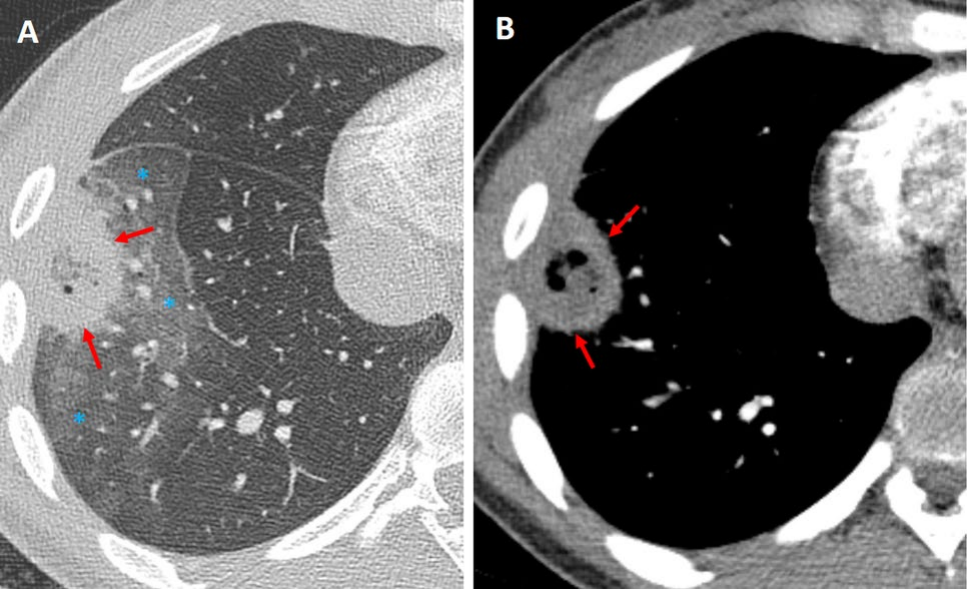
Atoll sign (arrows) in a patient with pulmonary paragonimiasis. Chest CT images with (**A**) lung window and (**B**) mediastinal window settings demonstrate a solitary, right lower lobe lateral basilar, subpleural irregular pulmonary opacity with the atoll sign (arrows) and surrounding ground-glass opacity (*). Note the adjacent pleural thickening

Pulmonary paragonimiasis, caused by the lung fluke *Paragonimus spp.*, can occasionally present with the RHS in association with pleural effusion/thickening or pneumothorax (Fig. 7) [6]. In this setting, granulomatous inflammation within the pulmonary parenchyma gives rise to the characteristic atoll appearance on imaging. Patients with paragonimiasis often have a history of ingesting raw or undercooked freshwater crustaceans and typically reside in or have traveled to endemic areas [3, 6]. In addition, laboratory evaluation may reveal peripheral eosinophilia and elevated serum IgE levels, features that are uncommon in bacterial or fungal infections.

### Inflammatory and autoimmune diseases

#### Organizing pneumonia

**Fig. 8:**
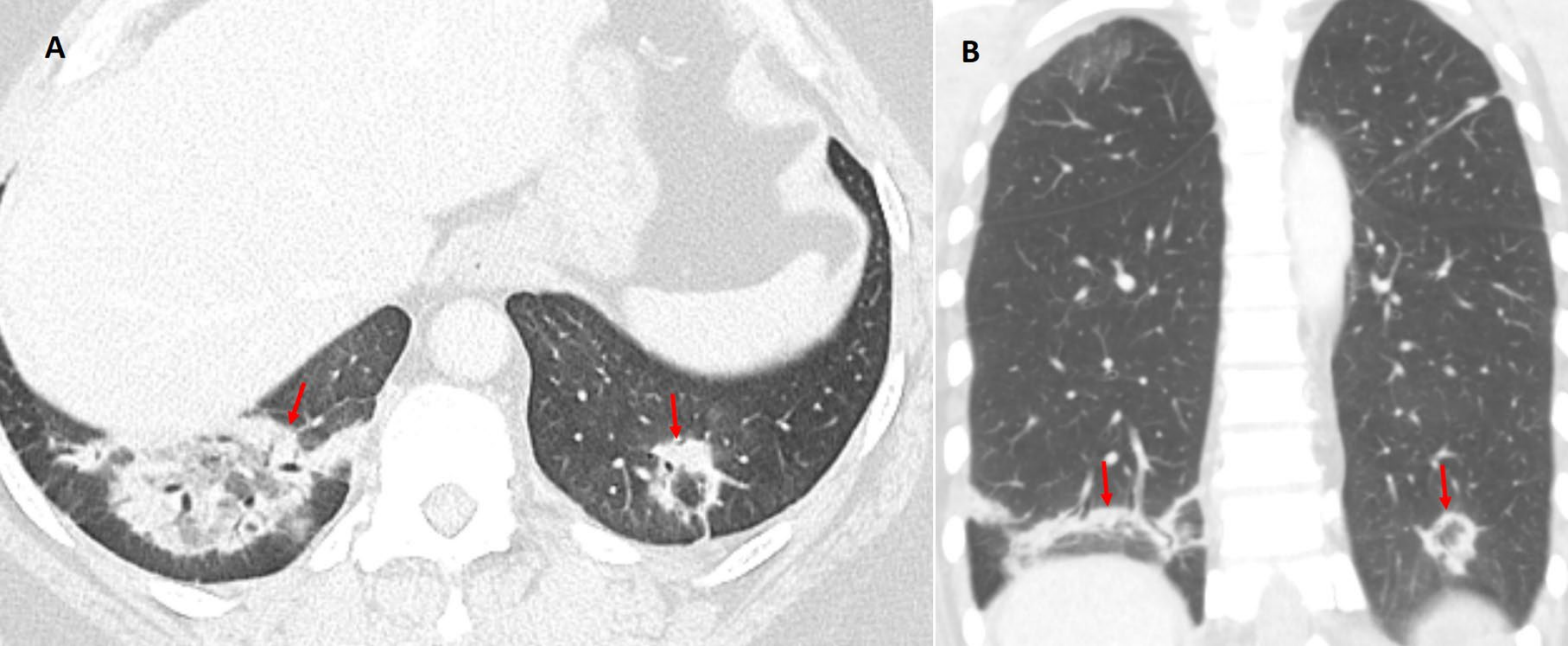
Examples of the reverse halo (atoll) sign (arrow) in a patient with breast cancer and organizing pneumonia (OP). The images demonstrate the characteristic peripheral and bronchovascular distribution of the pulmonary opacities

Organizing pneumonia (OP) is the prototypical condition linked to the RHS and often presents with bilateral, peripheral lesions in patients experiencing insidious symptoms such as cough or dyspnea. In OP, the central GGO reflects acute inflammatory and fibrinous exudates filling the alveoli, while the outer rim of consolidation represents organizing fibrosis. OP typically demonstrates a smooth, uniform rim of consolidation and may exhibit a migratory pattern on serial imaging, characteristics that are less common in infectious or neoplastic processes (Fig. 8) [7].

#### Vasculitis and autoimmune disorders

**Fig. 9:**
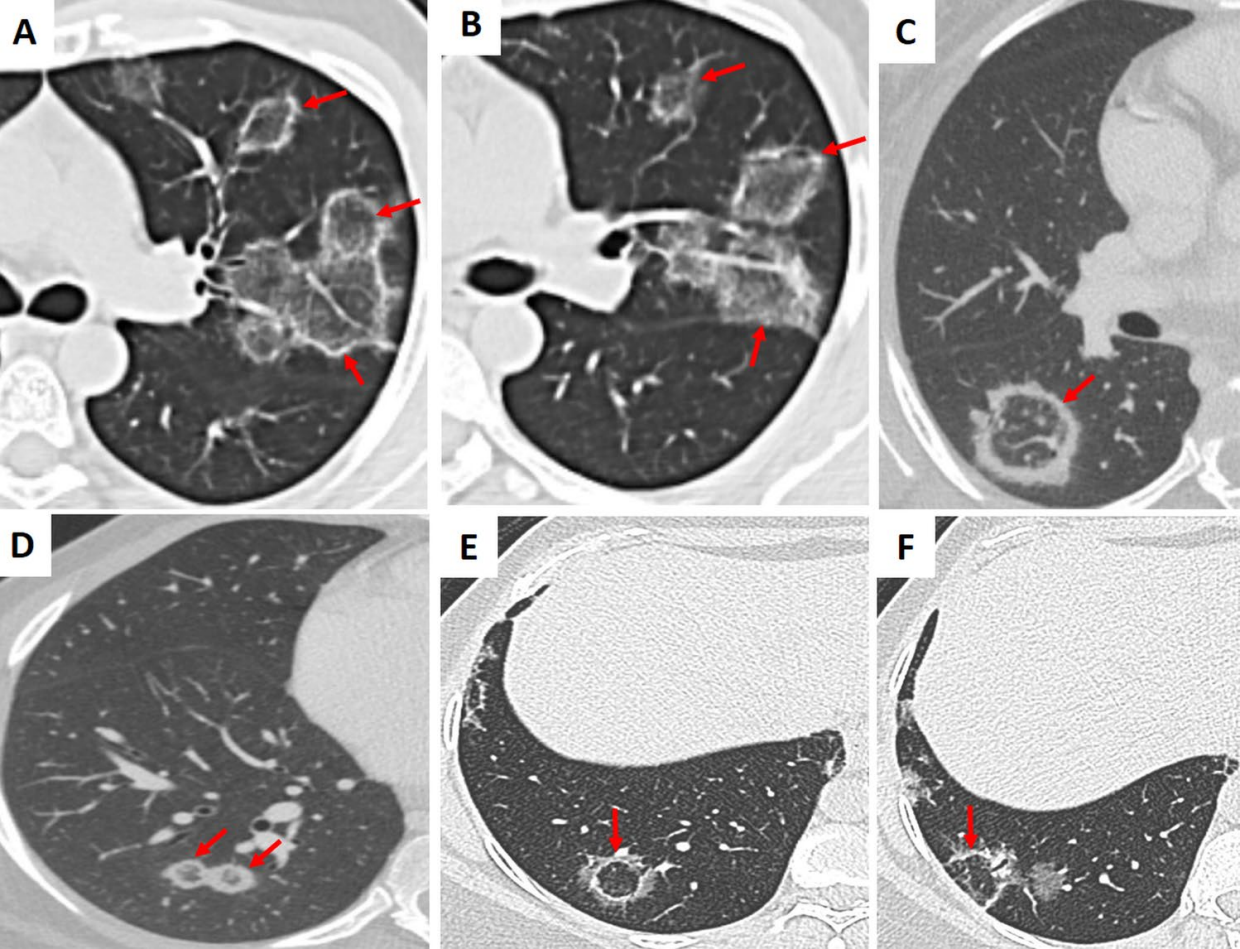
Examples of the atoll sign (arrows) in patients with (**A**, **B**) granulomatosis with polyangiitis, (**C**, **D**) systemic lupus erythematosus, and (**E**, **F**) rheumatoid arthritis. The images demonstrate the characteristic reverse halo sign in different autoimmune conditions, highlighting variations in distribution and associated pulmonary changes

**Fig. 10:**
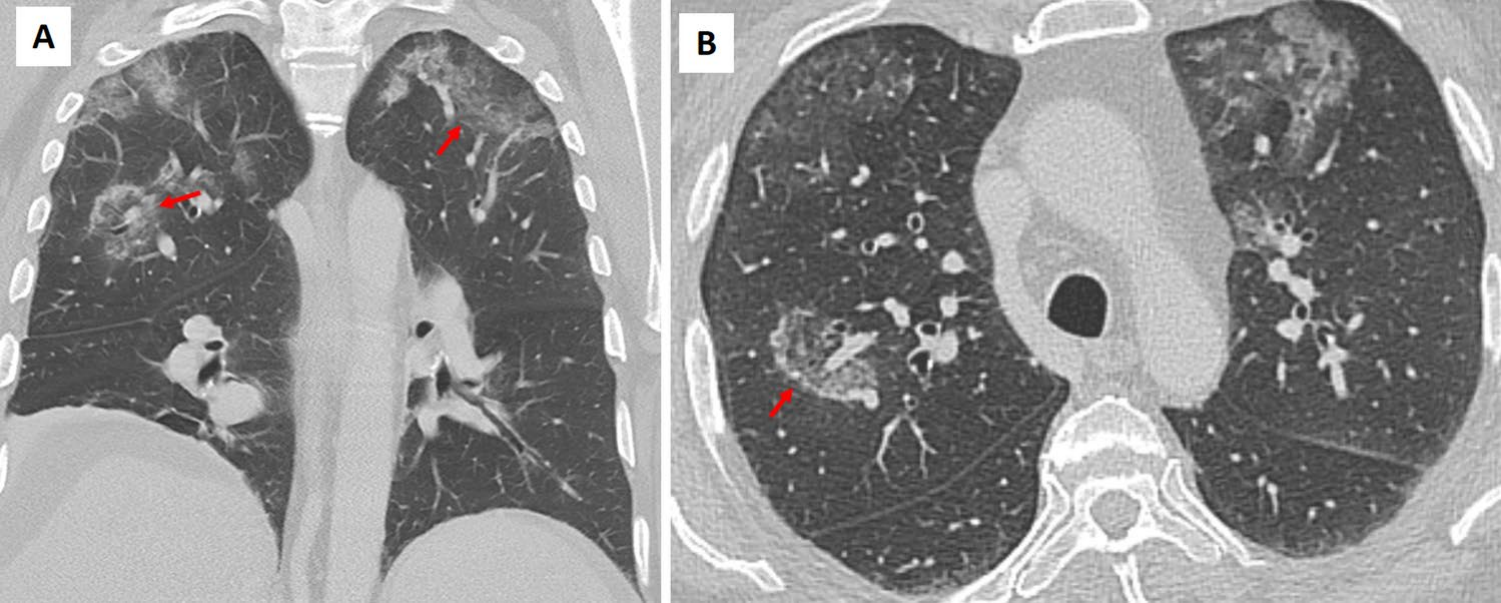
**A** Coronal and (**B**) axial chest CT images demonstrate upper lung-predominant ground-glass opacity (GGO) and the reverse halo sign (arrows) in a patient diagnosed with eosinophilic pneumonia. The patient had peripheral eosinophilia of 1264 eosinophils/μL (normal range ≥ 500 eosinophils/μL)

Autoimmune disorders such as granulomatosis with polyangiitis (GPA) and systemic lupus erythematosus (SLE) can present with the RHS. In GPA, the central ground-glass opacity reflects necrotizing granulomas or vasculitic lesions, while in SLE, lupus pneumonitis or alveolar hemorrhage produces a similar pattern (Figs. 9 and S3) (1–4). Eosinophilic pneumonia (EP), though rare, is characterized by alveolar inflammation with eosinophilic exudates and a peripheral rim of consolidation, accompanied by peripheral eosinophilia and elevated IgE levels (Fig. 10) [8]. Diagnosis is achieved by correlating imaging findings with clinical clues: for example, GPA often presents with systemic features such as sinusitis or renal involvement with positive ANCA, SLE is typically accompanied by malar rash, arthritis, and positive anti-dsDNA antibodies, and eosinophilic pneumonia usually manifests as upper lobe peripheral RHS lesions with marked eosinophilia.

#### Sarcoidosis

**Fig. 11:**
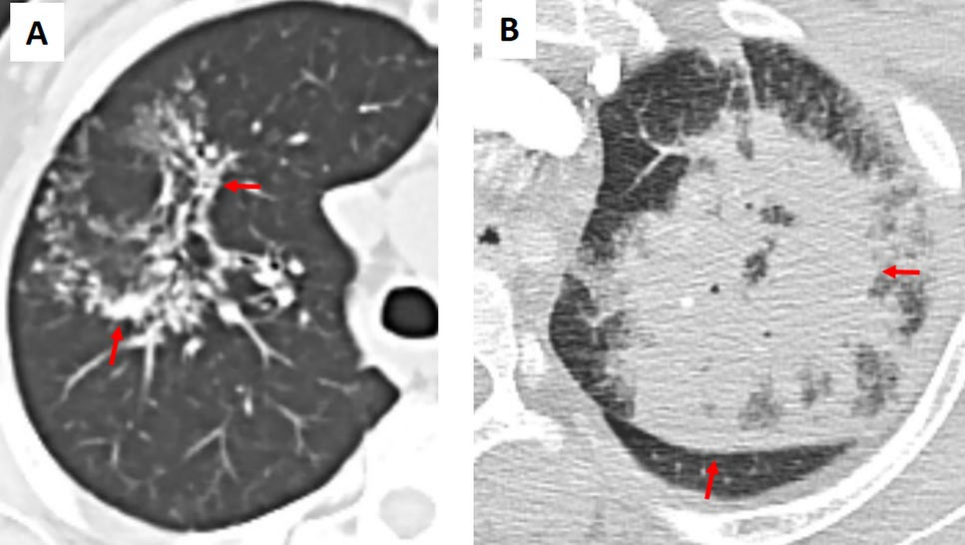
Reverse halo (atoll) sign (arrows) in two different patients with sarcoidosis. **A** Axial chest CT image shows multiple tiny coalescing nodules in the right upper lobe forming the atoll sign. **B** Axial chest CT image of a patient with alveolar sarcoidosis demonstrates left upper lobe consolidation with central ground-glass opacities consistent with the atoll sign

Sarcoidosis, a multisystem granulomatous disease, can sometimes produce coalescent nodules that form a RHS (Figs. 11). In this setting, the central area often represents active granulomatous inflammation, while the surrounding consolidative rim reflects an evolving granulomatous reaction. Radiologists can differentiate sarcoidosis from other causes of the atoll sign by identifying additional imaging features—such as bilateral hilar and mediastinal lymphadenopathy and a characteristic bronchovascular or perilymphatic distribution of nodules—that are typical of sarcoidosis but less common in organizing pneumonia, infections, or malignancies [1–4, 9]. Clinically, patients with sarcoidosis often exhibit systemic symptoms including fatigue, cough, and sometimes skin or ocular involvement, and may have supportive laboratory findings such as an elevated angiotensin-converting enzyme (ACE) level.

#### Lipoid pneumonia

Lipoid pneumonia results from the aspiration or inhalation of lipid-containing material, leading to the accumulation of lipid-laden macrophages within the alveolar spaces. In some cases, these lipid deposits produce a RHS on CT imaging [1, 2]. Patients with lipoid pneumonia often have a history of chronic aspiration, neurological deficits, or occupational exposure to oil-based substances, such as mineral oil or vegetable oil. On CT, lipoid pneumonia may exhibit areas of negative attenuation within the consolidation—reflecting the fat content—a feature that is typically absent in organizing pneumonia or infectious etiologies [1, 2]. Additionally, these patients usually lack systemic signs of infection.

### Vascular causes

#### Pulmonary Embolism (PE)

**Fig. 12:**
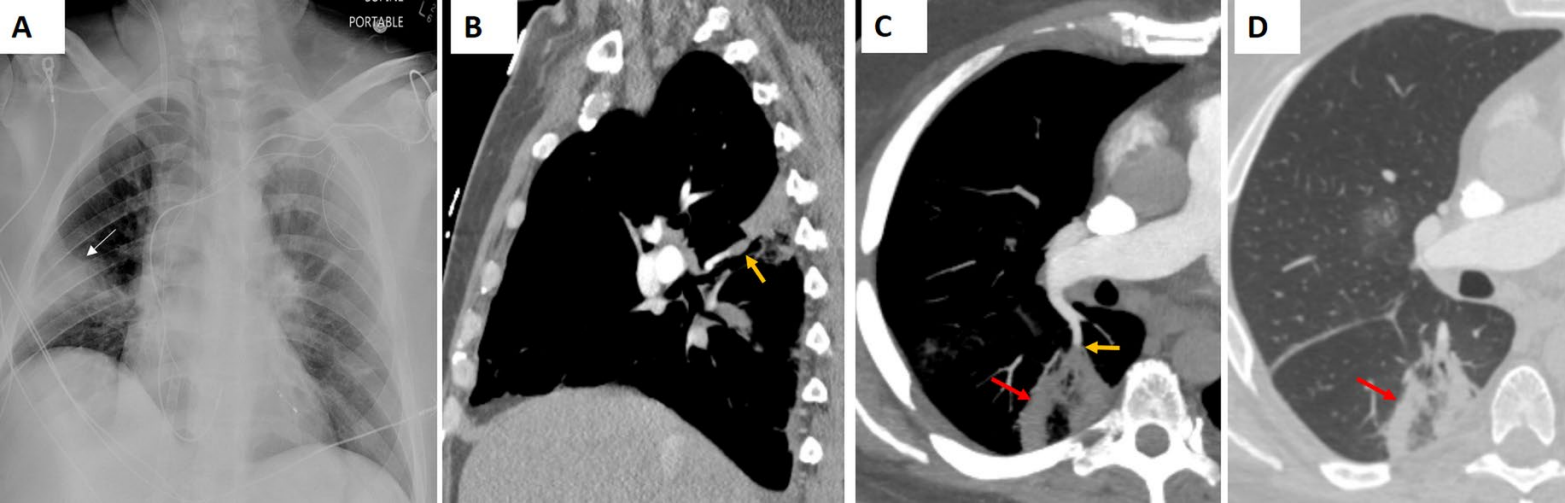
Atoll sign in a patient with pulmonary embolism. The patient had been monitored as an inpatient due to arrhythmia and developed chest pain over the past 24 h. **A** The frontal radiograph shows a peripheral, wedge-shaped opacity in the right mid-lung (white arrow). **B** Sagittal and (**C**, **D**) axial chest CT angiography images demonstrate pulmonary embolism (orange arrows) and a right lower lobe posterior basilar wedge-shaped pulmonary opacity with the atoll sign (red arrows), consistent with pulmonary infarction

PE can lead to wedge-shaped infarcts that manifest as the RHS, typically in the posterior or basal segments of the lung (Fig. 12). The central GGO represents ischemic lung parenchyma, and the surrounding consolidation reflects inflammatory hemorrhage or partial necrosis [3]. Clinically, patients with PE often present with acute onset dyspnea, pleuritic chest pain, and sometimes signs of right heart strain, and they usually have predisposing conditions such as deep vein thrombosis or hypercoagulable states. The PE-related RHS is characteristically wedge-shaped and peripherally distributed. Elevated D-dimer levels and findings on CT pulmonary angiography, such as direct visualization of intraluminal thrombus, further support the diagnosis [3, 4].

#### Septic pulmonary embolism

**Fig. 13:**
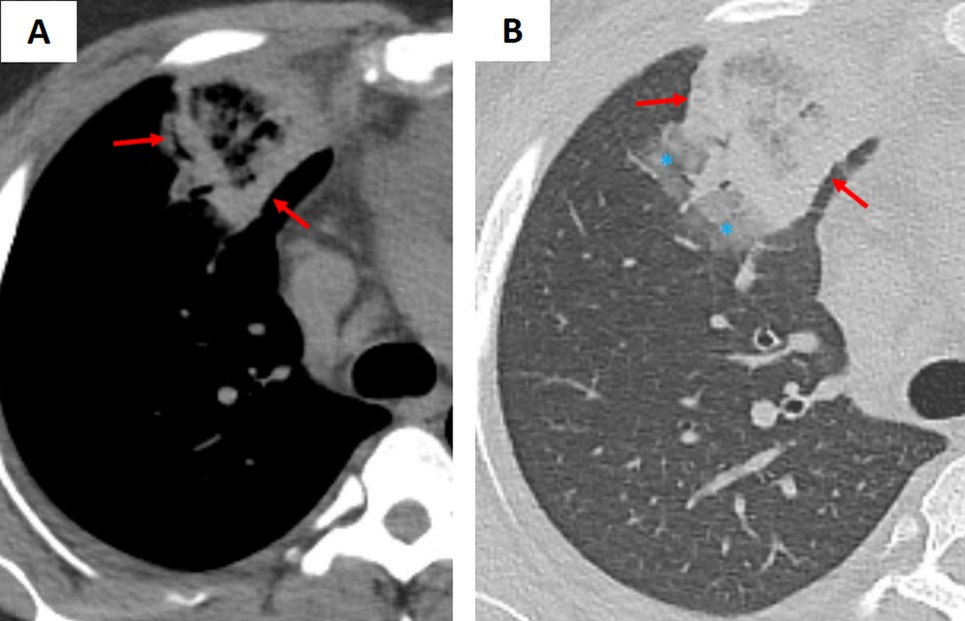
Atoll sign (arrows) in a patient with colon carcinoma and presented with cough and fever. The patient diagnosed with septic pulmonary embolism due to *Bacteroides fragilis* septicemia. Axial (**A**) mediastinal and (**B**) lung window CT images demonstrate a solitary, right upper lobe anterior wedge-shaped opacity with the atoll sign. Note the surrounding ground-glass opacity (*), indicative of associated inflammation, hemorrhage, or infarction

In septic pulmonary embolism the RHS may indicate infected infarcts (Fig. 13). These lesions are typically characterized by cavitation and the presence of multiple peripheral nodular opacities, which suggest an embolic process [1–4]. Unlike the smoother, more uniform appearance of OP or the solitary nature of certain neoplastic lesions, septic emboli often exhibit a heterogeneous and irregular morphology. Clinically, these patients usually present with signs of systemic infection, such as fever, elevated inflammatory markers, and positive blood cultures, as well as established risk factors like IV drug use or a history of endocarditis [3, 4].

### Malignant causes

#### Primary lung carcinoma

**Fig. 14:**
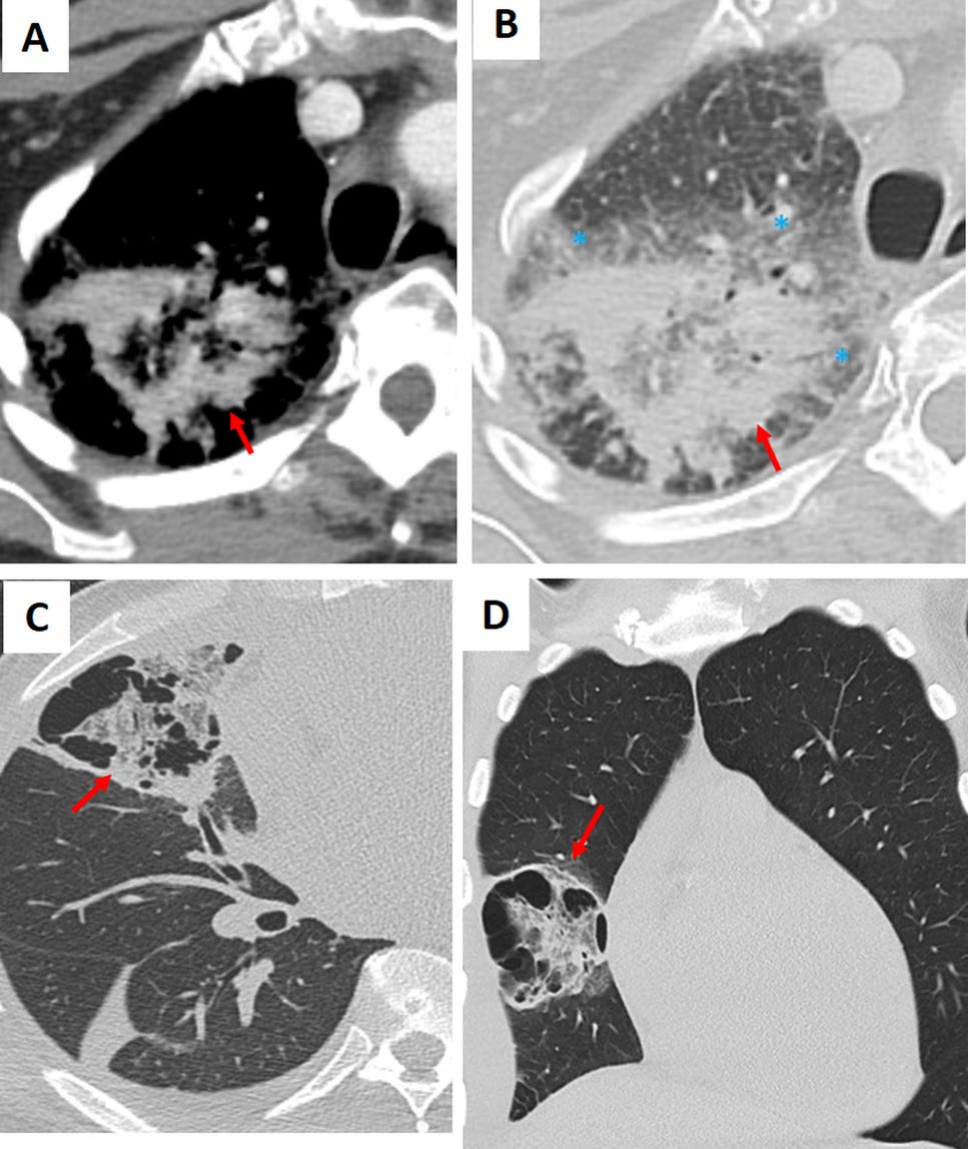
Reverse halo (atoll) sign (arrows) in two patients with primary lung carcinoma. **A**, **B** A patient with lung squamous cell carcinoma: Axial (**A**) mediastinal and (**B**) lung window CT images show an irregular, spiculated right upper lobe mass with the atoll sign (arrows). Note the surrounding ground-glass opacity (*), which may reflect an invasive component of the tumor. **C**, **D** A patient with invasive pulmonary adenocarcinoma: **C** Axial and (**D**) coronal chest CT images demonstrate a right middle lobe mass with the reverse halo sign and internal cystic airspaces (arrows)

Rarely primary lung cancers can exhibit the RHS (Fig. 14). In these malignancies, the central GGO often corresponds to tumor necrosis [10]. Lung cancer–related RHS is typically observed in patients with risk factors such as a significant smoking history, unexplained weight loss, and a more insidious clinical course without the acute infectious symptoms. On CT, malignant lesions may display irregular or spiculated margins, and they are more likely to be associated with adjacent lymphadenopathy or satellite nodules. Furthermore, unlike benign conditions that often improve with appropriate therapy, malignant lesions tend to persist or progress on follow-up imaging [3, 10].

#### Metastatic cancer

**Fig. 15:**
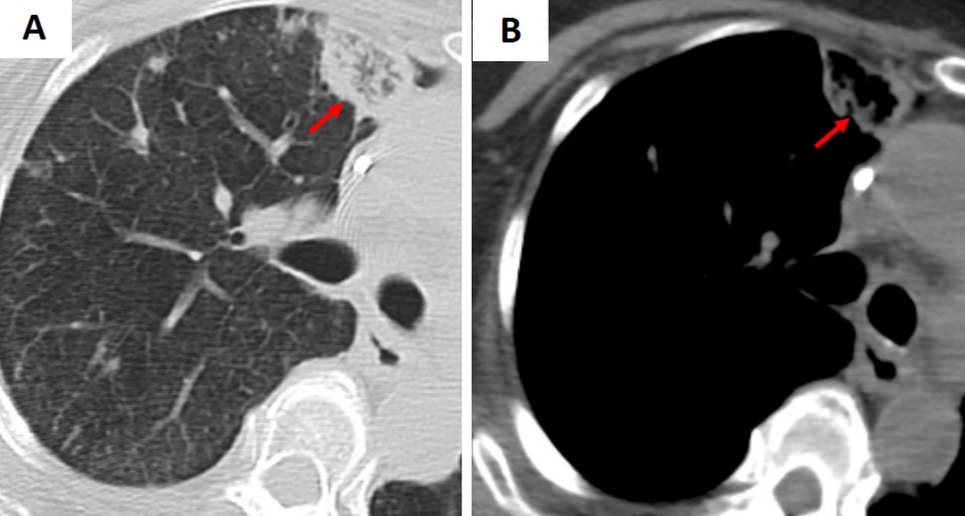
Reverse halo (atoll) sign in a patient with metastatic malignant melanoma. **A**, **B** Chest CT images demonstrate an irregular right upper lobe mass with the reverse halo (atoll) sign (arrows). Metastasis was confirmed via biopsy. Note the presence of additional pulmonary nodules suggestive of metastasis. The patient also had multiple hepatic metastases (not shown)

Metastatic lesions from malignancies can also create a RHS (Fig. 15) [4]. Metastasis-related RHS is often characterized by multiple irregular and heterogeneous lesions. In patients with a known history of cancer, the identification of such a pattern, along with associated findings like mediastinal or hilar lymphadenopathy, should prompt a comprehensive metastatic workup and staging [4, 11].

### Trauma and treatment related

#### Pulmonary contusion

**Fig. 16:**
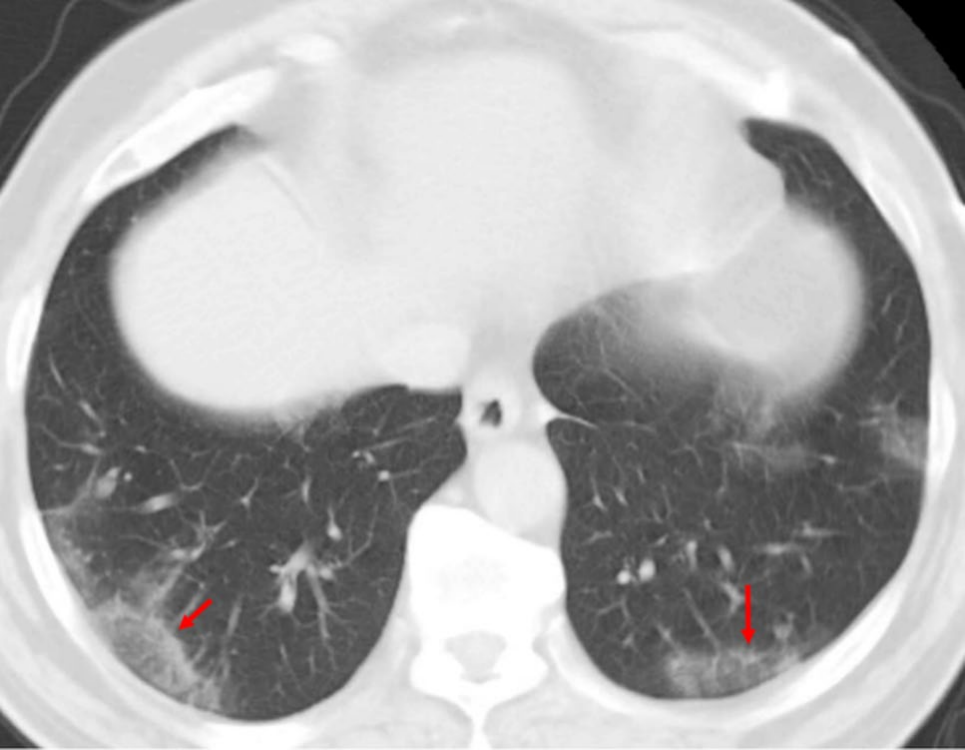
Reverse halo (atoll) sign in a patient with blunt chest trauma. Axial chest CT image demonstrates multiple subpleural pulmonary opacities with the atoll sign (arrows), likely reflecting pulmonary contusion. The patient also had non-displaced rib fractures (not shown)

Pulmonary contusions resulting from blunt chest trauma can produce a temporary peripheral RHS (Fig. 16). In these cases, the central GGO is due to alveolar hemorrhage and edema, while the peripheral rim of consolidation reflects the organizing inflammatory response to the injury. Pulmonary contusions are typically associated with a clear history of recent trauma and often present with concurrent clinical findings such as chest wall bruising or rib fractures. Moreover, these traumatic lesions characteristically resolve within a few days on follow-up imaging [1, 2].

#### Radiation-Induced Organizing Pneumonia (RIOP)

**Fig. 17:**
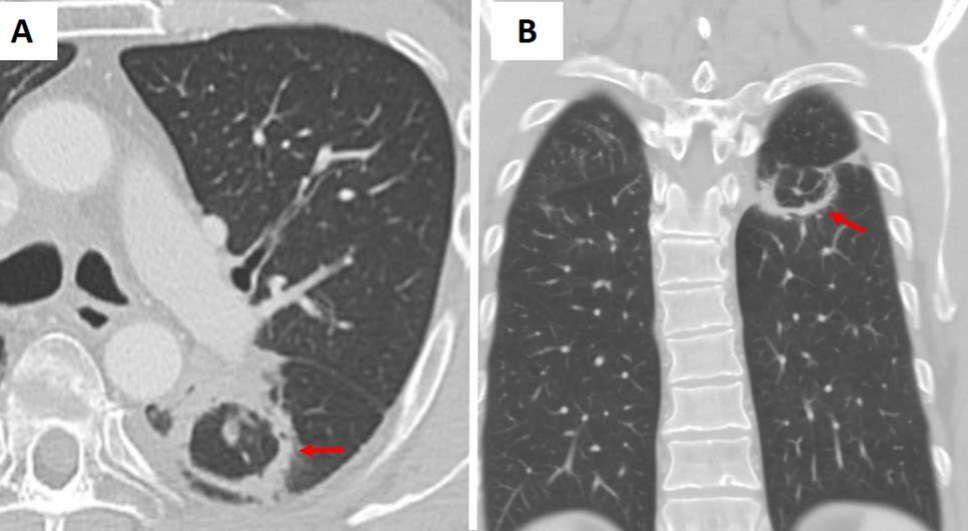
Reverse halo (atoll) sign in a patient with metastatic left lung squamous cell carcinoma who underwent hilar radiotherapy and developed radiation-induced organizing pneumonia (RIOP). **A** Axial and (**B**) coronal chest CT images demonstrate a pulmonary opacity with the atoll sign (arrows) adjacent to the irradiated left hilum

Patients who have undergone thoracic radiotherapy may develop radiation-induced organizing pneumonia (RIOP). In RIOP, the RHS can appear both within and outside the radiation field, reflecting a widespread inflammatory and fibrotic response in susceptible lung segments (Figs. 17 and S4) [1, 4, 12]. RIOP often exhibits a more uniform and well-demarcated pattern with accompanying diffuse GGO. Clinical correlation is critical; a history of recent radiotherapy combined with a gradual onset of respiratory symptoms and radiologic findings that improve with corticosteroid therapy strongly supports RIOP.

#### Drug-related pneumonitis

**Fig. 18:**
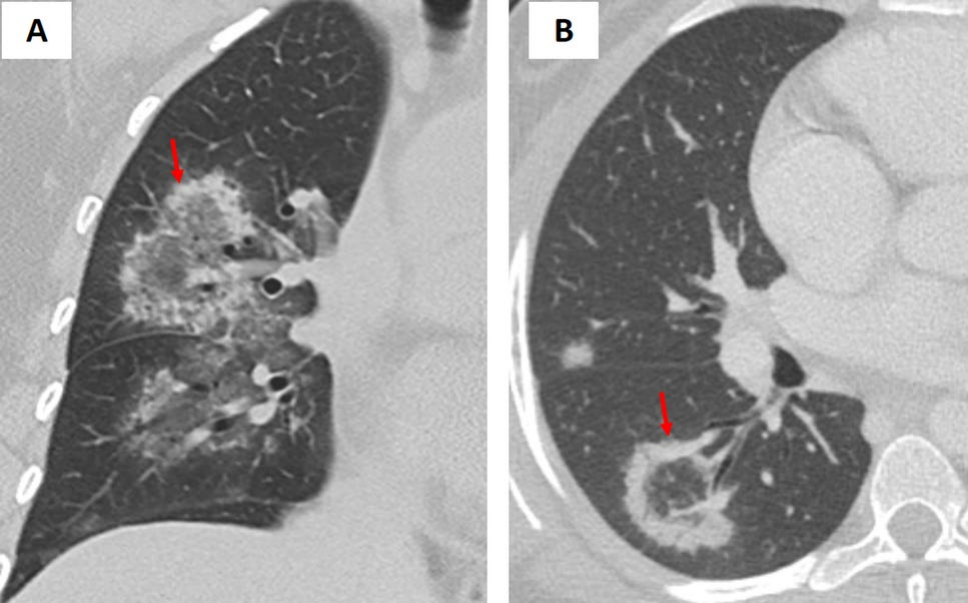
Atoll sign in a patient with malignant melanoma and immunotherapy-induced pneumonitis. **A** Coronal and (**B**) axial chest CT images demonstrate multiple peribronchial pulmonary opacities with the atoll sign (arrows). The causative agent was discontinued, and the opacities subsequently resolved

Certain medications, particularly checkpoint inhibitors and chemotherapeutic drugs, can provoke pneumonitis that displays a RHS (Fig. 18) [13]. Drug-related pneumonitis is typically characterized by a clear temporal relationship with drug administration, and patients often lack systemic infectious symptoms such as fever or leukocytosis. Moreover, the imaging findings in drug-induced pneumonitis usually occur in the context of diffuse or patchy GGO with a RHS [13, 14]. Improvement or stabilization of these lesions on follow-up imaging after discontinuation of the offending drug and initiation of corticosteroid therapy further supports a diagnosis of drug-related pneumonitis.

#### Lung nodule focal therapy and ablation

Ablative procedures—such as radiofrequency ablation (RFA), microwave ablation (MWA), or cryoablation—can produce a RHS in the treated region, characterized by central coagulative necrosis surrounded by an inflammatory rim [15]. By correlating procedural history with serial CT findings, radiologists can confidently attribute the observed atoll sign to benign post-treatment changes, thereby avoiding unnecessary interventions.

## Conclusion

The reverse halo sign is a key radiological feature that can be seen across a wide range of lung conditions. Accurate interpretation, through correlation with clinical history and diagnostic findings, is essential for timely diagnosis, guiding appropriate treatment, and ultimately improving patient outcomes (Table 1 and 2).

**Table 1:** The imaging and key diagnostic features of the causes of the reverse halo (atoll) sign

Cause / Condition	Imaging Findings	Key Diagnostic Clues
Infectious: Bacterial Pneumonia	Central ground-glass opacity (GGO) surrounded by consolidation, often appearing in the organizing phase of lung injury	Recent or resolving pneumonia; *Staphylococcus aureus* commonly implicated; improvement with targeted antibiotic therapy or supportive treatment
Infectious: Fungal (Mucormycosis, Aspergillosis)	"Bird’s nest" variant of the atoll sign with central necrotic tissue (GGO) and a peripheral rim of inflammation or fibrosis	Common in immunocompromised or diabetic patients; urgent, aggressive antifungal therapy required for mucormycosis; serologic or microbiological tests aid differentiation from other fungi
Infectious: Viral Pneumonia (COVID-19, Influenza, CMV, RSV)	Ground-glass central opacity indicative of viral lung injury, with a consolidative "ring" reflecting immune response; may appear in later or resolving phases of infection	Clinical and laboratory findings of viral infection; differentiation from bacterial pneumonia and other interstitial processes important; post-inflammatory fibrosis can manifest as a reverse halo
Infectious: Parasitic (Pulmonary Paragonimiasis)	Reverse halo sign sometimes accompanied by pleural effusion or pneumothorax; central granulomatous changes surrounded by peripheral consolidation	Relevant travel or endemic exposure history; serology or stool examination for *Paragonimus* species; differentiation from other granulomatous diseases (e.g., tuberculosis, sarcoidosis)
Inflammatory / Autoimmune: Organizing Pneumonia (OP)	Classic reverse halo sign with a central area of GGO representing acute inflammatory exudates, surrounded by organizing fibrosis or consolidation. Migratory opacities	Typically responds to corticosteroids; may mimic infection or malignancy; biopsy or follow-up often needed for confirmation
Inflammatory / Autoimmune: Granulomatosis with Polyangiitis (GPA), SLE	Central necrotic or vasculitic process encircled by inflammatory tissue; can resemble infection on imaging	Multi-system involvement; serologic markers (e.g., ANCA in GPA, ANA in SLE); prompt immunosuppressive therapy often required after excluding infection
Inflammatory / Autoimmune: Sarcoidosis	Coalescent nodules producing a reverse halo pattern with central necrosis or active granuloma and surrounding inflammatory changes	Typical association with bilateral hilar lymphadenopathy; biopsy may be needed to rule out infection or malignancy; often a chronic, systemic disease course
Inflammatory / Autoimmune: Lipoid Pneumonia	Reverse halo pattern due to lipid-laden macrophages in alveolar spaces; GGO often predominates in the center with a rim of denser consolidation	History of chronic aspiration or exposure to oil-based substances; excludes typical organisms; may require biopsy for definitive diagnosis
Vascular: Pulmonary Embolism and Infarction	Wedge-shaped, posterior basal lesion with central GGO indicating ischemic lung, bordered by hemorrhage or inflammatory consolidation forming a reverse halo	Risk factors such as deep vein thrombosis, atrial fibrillation, hypercoagulable states; confirmation with CT pulmonary angiography; must distinguish from infection or malignancy in certain cases
Vascular: Septic Pulmonary Embolism	Similar wedge-shaped or nodular pattern; may be cavitary due to infected emboli; reverse halo sign indicates infarction with an infected rim	Seen in IV drug use or infective endocarditis; multiple peripheral nodules often present; clinical correlation (fever, positive blood cultures) essential
Malignant: Primary Lung Carcinoma	Reverse halo commonly seen in larger or cavitary tumors, such as squamous cell carcinoma; central necrosis or hemorrhage forms the GGO center with a tumor or inflammatory rim	Biopsy necessary to differentiate from benign entities; consider risk factors (smoking history, unexplained weight loss, hemoptysis)
Malignant: Metastatic Cancer	Central necrosis within metastatic lesions surrounded by peripheral tumor infiltration or reactive fibrosis, creating the atoll sign	Often identified in patients with known primary malignancies; PET-CT or histological confirmation guides further staging and treatment
Trauma: Pulmonary Contusion	Reverse halo appearance in acute contusions due to alveolar hemorrhage (central GGO) and organizing inflammatory changes (rim of consolidation), typically transient	History of blunt chest trauma; radiologic improvement usually seen in a few days; distinguished from infectious pneumonia based on clinical improvement and short interval follow-up
Radiation-Induced Organizing Pneumonia (RIOP)	Reverse halo sign may appear weeks to months after radiation therapy, affecting both irradiated and adjacent lung segments; mimics organizing pneumonia	Recent radiotherapy history; improvement with corticosteroids; important to differentiate from tumor recurrence
Drug-Related Pneumonitis (Immunotherapy, Chemotherapy)	Inflammatory lung changes manifesting as the RHS; central GGO with outer consolidation reflecting drug-induced alveolar damage	Strong association with recent drug exposure; discontinuation of the offending agent and corticosteroid therapy often required; must exclude infection or malignancy in oncology patients
Lung Nodule Focal Therapy and Ablation	Reverse halo sign in the ablated region where central necrosis (coagulative tumor destruction) is encircled by an inflammatory rim; can persist for weeks to months	Common post-RFA, MWA, or cryoablation; essential to distinguish from infection or residual/recurrent tumor; correlating with procedure timeline and follow-up imaging helps avoid misdiagnosis

*RHS* Reverse Halo Sign, *GGO* Ground-Glass Opacity, *CT* Computed Tomography, *IV* Intravenous, *ANCA* Anti-Neutrophil Cytoplasmic Antibodies, *ACE* Angiotensin-Converting Enzyme, *IgE* Immunoglobulin E, *PE* Pulmonary Embolism, *SLE* Systemic Lupus Erythematosus, *GPA* Granulomatosis with Polyangiitis, *RIOP* Radiation-Induced Organizing Pneumonia, *MWA* Microwave Ablation, *RFA* Radiofrequency Ablation

**Table 2:** This table provides a structured approach to evaluating the reverse halo sign (RHS) by categorizing conditions based on their radiological features, anatomical distribution, and associated clinical clues

Category	Feature	Conditions	Diagnostic/Clinical Clues
Multiplicity	Single RHS	Pulmonary infarction (PE), Organizing pneumonia, Primary lung carcinoma, Pulmonary paragonimiasis, contusion, RIOP	Acute onset (PE), steroid responsiveness (OP, RIOP), trauma/radiotherapy history
Multiplicity	Multiple RHS	Viral pneumonia (e.g., COVID-19), Septic emboli, Metastatic cancer, Drug-induced pneumonitis	Recent viral exposure, systemic infection signs, known malignancy, recent drug exposure
Anatomic Location	Upper Lung Predominance	Sarcoidosis, Tuberculosis, Eosinophilic pneumonia	Hilar lymphadenopathy (Sarcoidosis), eosinophilia (Eosinophilic pneumonia), travel/endemic exposure (TB)
Anatomic Location	Lower Lung Zones	Pulmonary embolism, Pulmonary infarction, Viral pneumonia (COVID-19), Organizing pneumonia	Acute symptoms (PE/infarction), recent infection (COVID-19), clinical improvement with corticosteroids
Distribution	Peripheral Lung	Organizing pneumonia, COVID-19, Pulmonary infarction, Eosinophilic pneumonia, Pulmonary contusion	Recent illness history (COVID-19), peripheral eosinophilia (EP), recent trauma (contusion)
Distribution	Central/Bronchovascular	Drug-induced pneumonitis, Radiation-induced OP	Recent drug exposure (Drug-induced pneumonitis), recent radiotherapy (RIOP)
Rim Characteristics	Smooth, Uniform	Organizing pneumonia, Vasculitis, Radiation-induced OP, Bacterial pneumonia (organizing phase)	Improvement with steroids (OP, RIOP, vasculitis), clinical signs of infection (Bacterial pneumonia)
Rim Characteristics	Irregular/Nodular	Fungal infections, Metastatic disease, Primary lung carcinoma, Septic emboli	Immunocompromised status (fungal), known malignancy, systemic infection signs (septic emboli)
Associated Features	Pleural Effusion	Bacterial pneumonia, Parasitic infections	Infection signs, travel or dietary exposure (Parasitic infections)
Associated Features	Cavitation	Septic emboli, Primary lung carcinoma	Endocarditis risk (septic emboli), smoking history, unexplained weight loss (carcinoma)
Associated Features	Lymphadenopathy	Malignancy, Sarcoidosis	Known malignancy history, systemic symptoms, elevated serum ACE (sarcoidosis)

**Infectious Causes:** If the patient presents with signs of infection (fever, cough) or has an immunocompromised status, consider bacterial pneumonia (with a smooth rim), fungal infections (especially with a “bird’s nest” appearance), viral pneumonia (notably in the setting of COVID-19 or influenza), or parasitic infections (with relevant exposure history).

**Inflammatory / Autoimmune Causes:** In patients with subacute symptoms or systemic features, organize the differential around organizing pneumonia, autoimmune vasculitides (GPA, SLE), sarcoidosis, or lipoid pneumonia (with aspiration history).

**Vascular Causes:** Wedge-shaped, peripheral lesions in patients with risk factors for thrombosis point toward pulmonary embolism/infarction or septic emboli (if infection is present).

**Malignant Causes:** In patients with risk factors such as a smoking history or known cancer, consider primary lung carcinoma or metastasis.

**Trauma / Treatment-Related Causes:** A history of recent chest trauma, radiotherapy, drug exposure, or lung ablation will steer you toward diagnoses like pulmonary contusion, radiation-induced organizing pneumonia, drug-related pneumonitis, or post-ablation changes.

**Correlate with Clinical and Laboratory Data:** The imaging features must be integrated with the patient’s clinical history, laboratory tests, and other imaging findings to refine the diagnosis.

## Supplementary Information

Below is the link to the electronic supplementary material.

Fig. 19 Supplementary figure 1 Figure S1: (A) Initial axial chest CT image demonstrate a focal consolidation within the right lower lobe in a patient with COPD presenting with fever and cough. Sputum culture confirmed *Staphylococcus aureus* infection. (B) Axial chest CT image obtained three weeks later reveals the development of the reverse halo (atoll) sign at the site of the prior consolidation. Note the resolved additional pulmonary opacities.

High resolution image (TIF 3.90 MB)

Fig. 20 Supplementary figure 2 Figure S2: Reverse halo (atoll) sign (arrows) in a patient with COVID-19. (A) Frontal chest radiograph shows basilar and peripheral predominant multifocal pulmonary opacities. (B, C) Axial and coronal chest CT images demonstrate multiple areas of ground-glass opacity (GGO) surrounded by a peripheral rim of consolidation, characteristic of the reverse halo sign.

High resolution image (TIF 3.63 MB)

Fig. 21 Supplementary figure 2 Figure S3: (A) Initial axial chest CT image demonstrates multifocal pulmonary opacities with the reverse halo sign (RHS) in a patient with granulomatosis with polyangiitis (GPA). (B) Axial chest CT image obtained eight weeks later reveals a decrease in pulmonary opacities with residual RHS (arrows).

High resolution image (TIF 2.30 MB)

Fig. 22 Supplementary figure 2 Figure S4: Reverse halo (atoll) sign in a patient with metastatic right lung squamous cell carcinoma who underwent right hilar radiotherapy and subsequently developed radiation-induced organizing pneumonia (RIOP) within the left lower lobe. (A–C) Axial chest CT images demonstrate a pulmonary opacity with the reverse halo (atoll) sign (red arrows) located outside the radiation field of the irradiated left hilum (orange arrows).

High resolution image (TIF 2.69 MB)

## Data Availability

Data sharing not applicable – no new data generated in this article.
