# Supplementary figures and images for: The reverse halo sign in emergency chest CT: A diagnostic guide across diverse pulmonary conditions
Source: Emerg Radiol. 2025 Apr 15;32(3):435–45. doi: 10.1007/s10140-025-02337-2 (PMC12133904; doi:10.1007/s10140-025-02337-2)

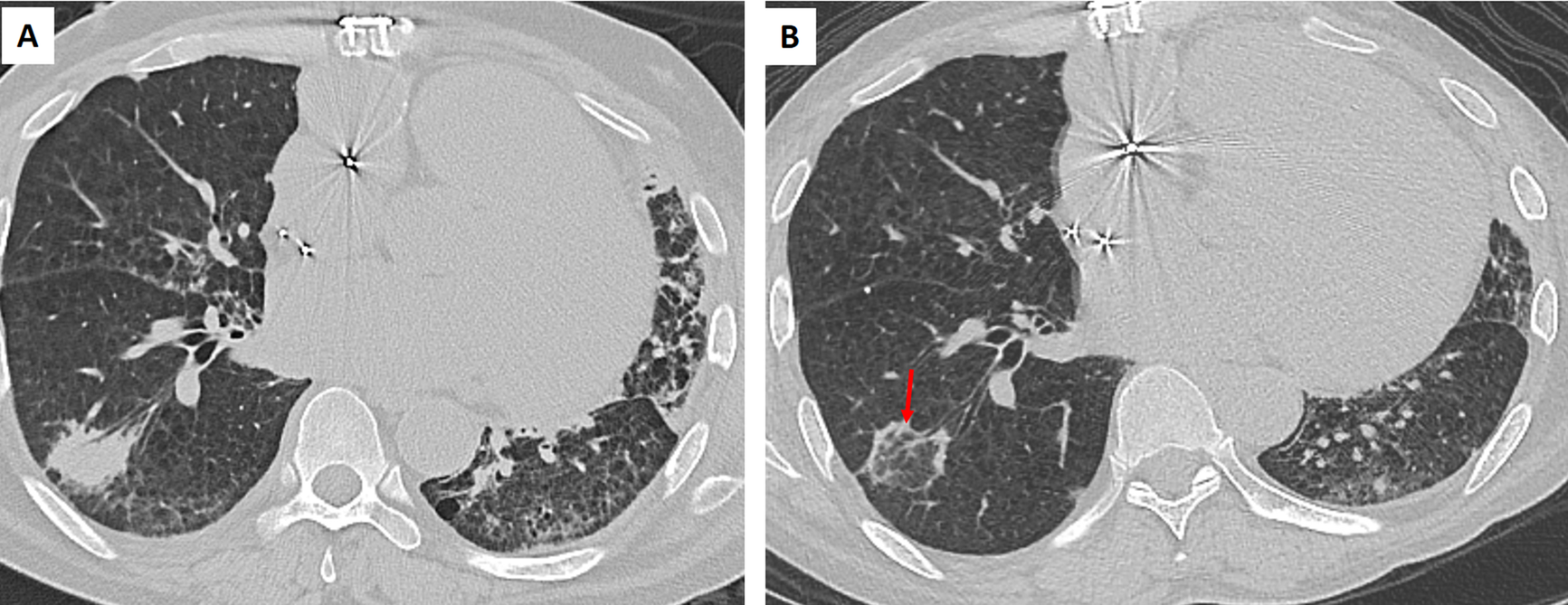

Supplement: Supplementary file 1 — Supplementary figure 1 Figure S1: (A) Initial axial chest CT image demonstrate a focal consolidation within the right lower lobe in a patient with COPD presenting with fever and cough. Sputum culture confirmed Staphylococcus aureus infection. (B) Axial chest CT image obtained three weeks later reveals the development of the reverse halo (atoll) sign at the site of the prior consolidation. Note the resolved additional pulmonary opacities. [file 10140_2025_2337_Fig19_ESM.png]

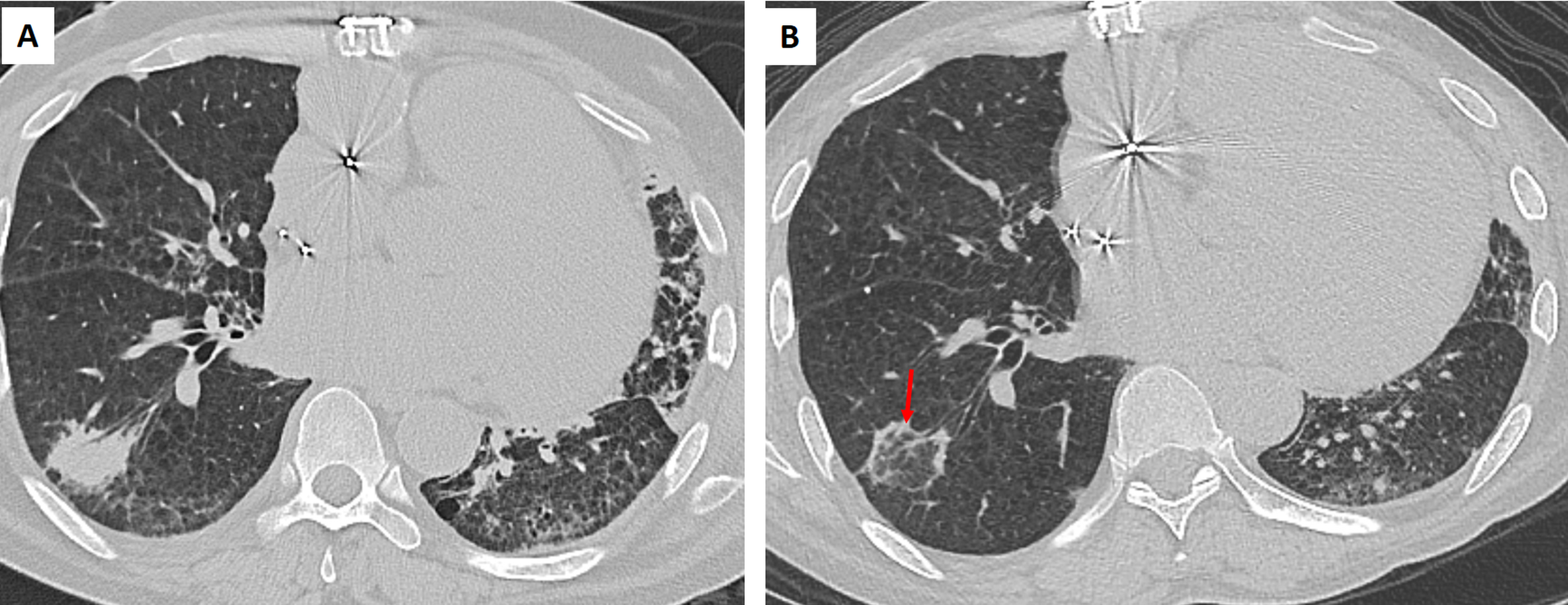

Supplement: Supplementary file 2 — High resolution image (TIF 3.90 MB) [file 10140_2025_2337_MOESM1_ESM.tif]

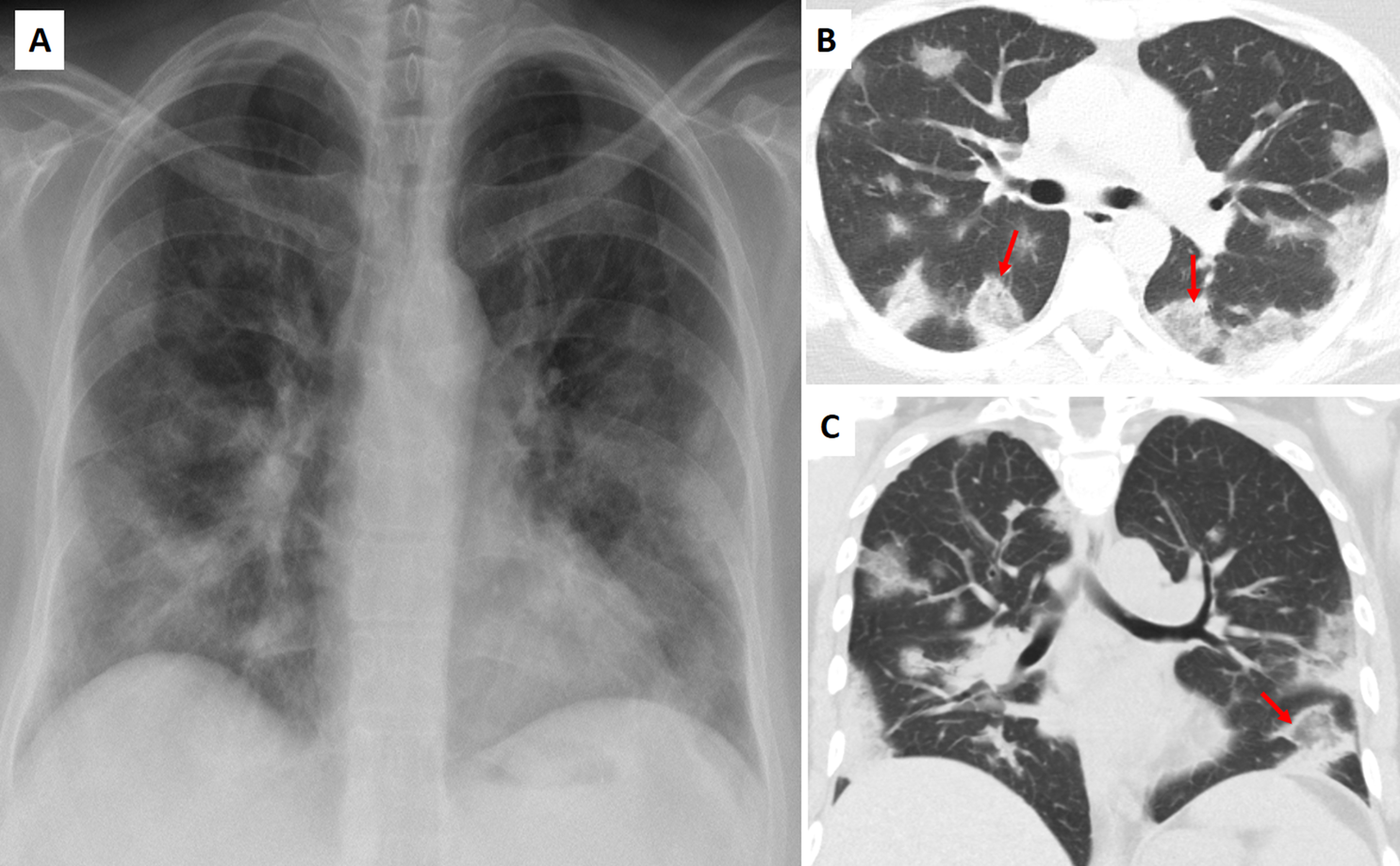

Supplement: Supplementary file 3 — Supplementary figure 2 Figure S2: Reverse halo (atoll) sign (arrows) in a patient with COVID-19. (A) Frontal chest radiograph shows basilar and peripheral predominant multifocal pulmonary opacities. (B, C) Axial and coronal chest CT images demonstrate multiple areas of ground-glass opacity (GGO) surrounded by a peripheral rim of consolidation, characteristic of the reverse halo sign. [file 10140_2025_2337_Fig20_ESM.png]

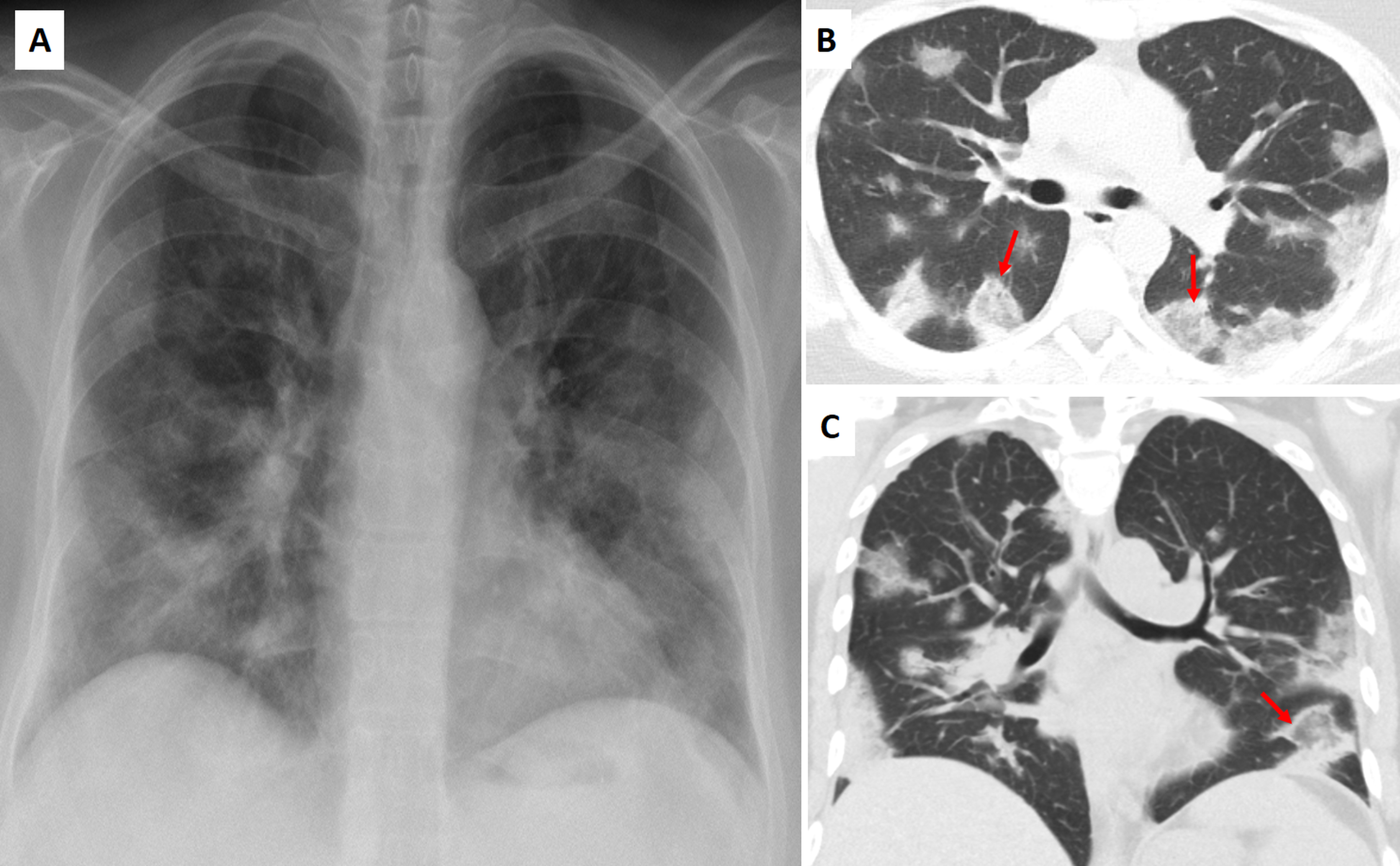

Supplement: Supplementary file 4 — High resolution image (TIF 3.63 MB) [file 10140_2025_2337_MOESM2_ESM.tif]

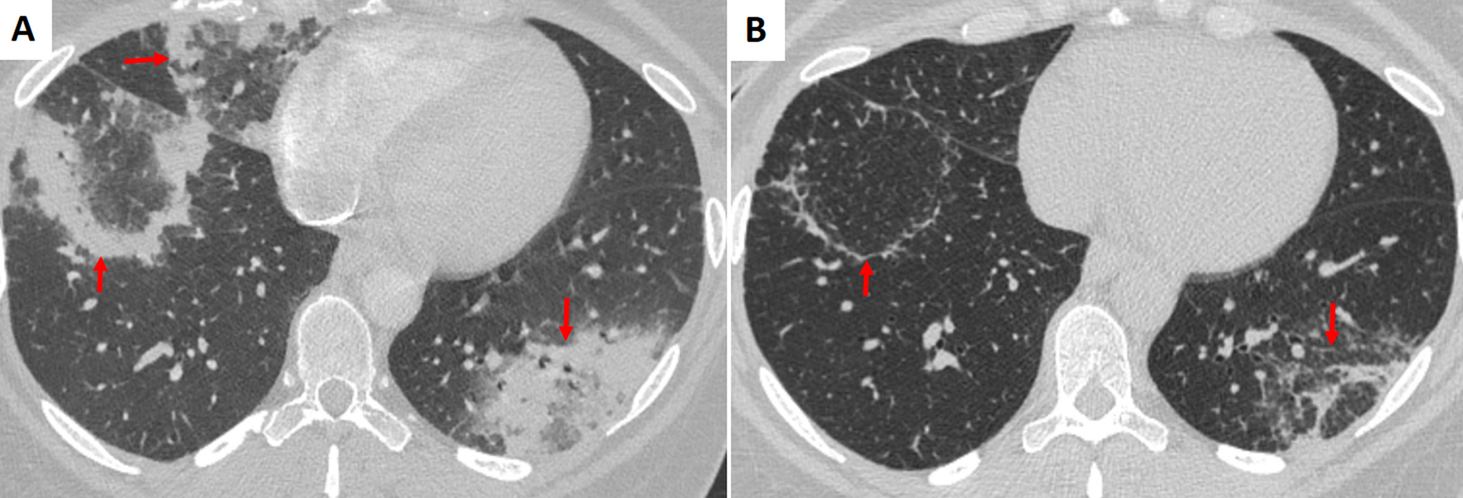

Supplement: Supplementary file 5 — Supplementary figure 2 Figure S3: (A) Initial axial chest CT image demonstrates multifocal pulmonary opacities with the reverse halo sign (RHS) in a patient with granulomatosis with polyangiitis (GPA). (B) Axial chest CT image obtained eight weeks later reveals a decrease in pulmonary opacities with residual RHS (arrows). [file 10140_2025_2337_Fig21_ESM.png]

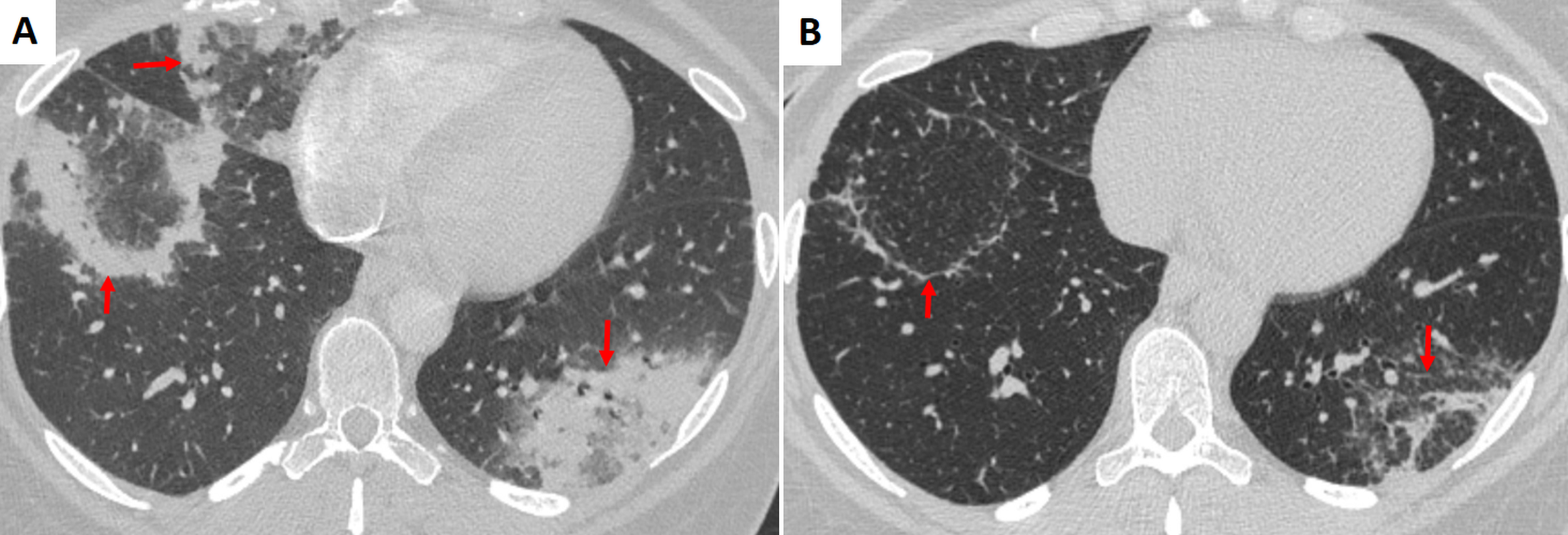

Supplement: Supplementary file 6 — High resolution image (TIF 2.30 MB) [file 10140_2025_2337_MOESM3_ESM.tif]

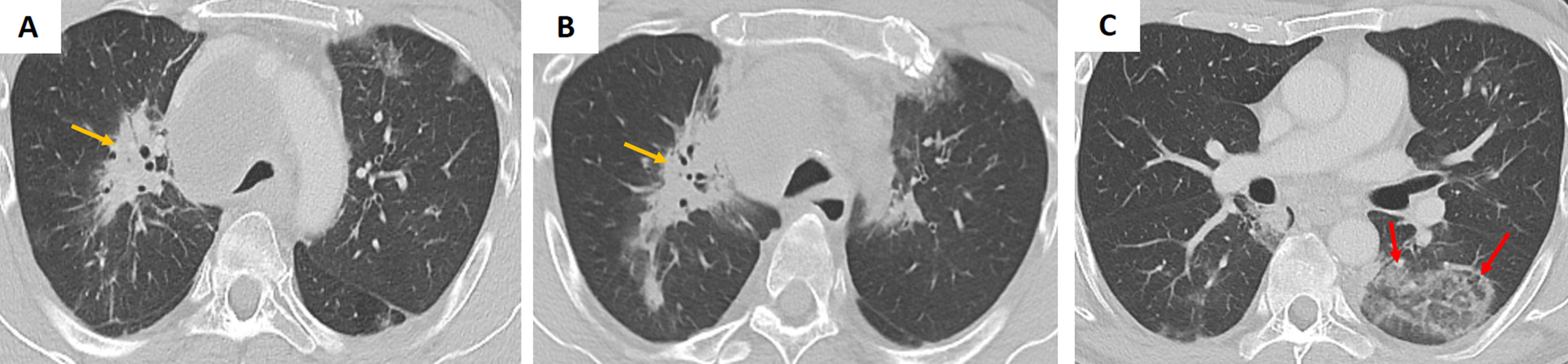

Supplement: Supplementary file 7 — Supplementary figure 2 Figure S4: Reverse halo (atoll) sign in a patient with metastatic right lung squamous cell carcinoma who underwent right hilar radiotherapy and subsequently developed radiation-induced organizing pneumonia (RIOP) within the left lower lobe. (A–C) Axial chest CT images demonstrate a pulmonary opacity with the reverse halo (atoll) sign (red arrows) located outside the radiation field of the irradiated left hilum (orange arrows). [file 10140_2025_2337_Fig22_ESM.png]

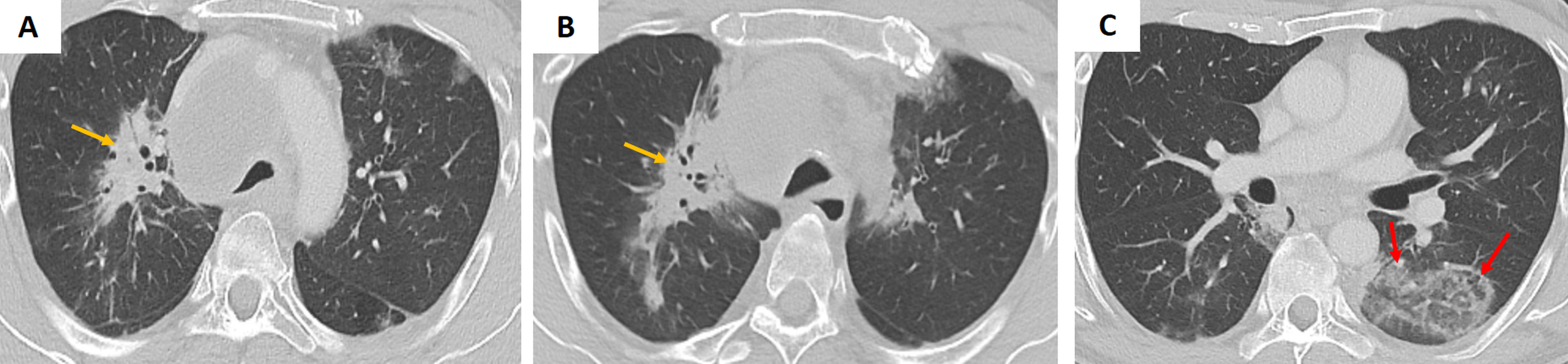

Supplement: Supplementary file 8 — High resolution image (TIF 2.69 MB) [file 10140_2025_2337_MOESM4_ESM.tif]
